# Whole Genome Sequencing of a Canadian Bovine Gammaherpesvirus 4 Strain and the Possible Link between the Viral Infection and Respiratory and Reproductive Clinical Manifestations in Dairy Cattle

**DOI:** 10.3389/fvets.2017.00092

**Published:** 2017-06-16

**Authors:** Carl A. Gagnon, Carolina Kist Traesel, Nedzad Music, Jérôme Laroche, Nicolas Tison, Jean-Philippe Auger, Sanela Music, Chantale Provost, Christian Bellehumeur, Levon Abrahamyan, Susy Carman, Luc DesCôteaux, Steve J. Charette

**Affiliations:** ^1^Swine and Poultry Infectious Diseases Research Center (CRIPA) and Groupe de recherche sur les maladies infectieuses en production animale (GREMIP), Faculté de médecine vétérinaire (FMV), Université de Montréal, St-Hyacinthe, QC, Canada; ^2^Institut de biologie intégrative et des systèmes (IBIS), Université Laval, Québec, QC, Canada; ^3^Département des Sciences cliniques, FMV, Université de Montréal, St-Hyacinthe, QC, Canada; ^4^Animal Health Laboratory, Laboratory Services Division, University of Guelph, Guelph, ON, Canada; ^5^Département de biochimie, de microbiologie et de bio-informatique, Université Laval, Québec, QC, Canada; ^6^Centre de recherche de l’Institut universitaire de cardiologie et de pneumologie de Québec, Québec, QC, Canada

**Keywords:** bovine gammaherpesvirus 4, long unique coding region, phylogenetic analysis, seroprevalence, cattle infectious diseases

## Abstract

Bovine gammaherpesvirus 4 (BoHV-4) is a herpesvirus widespread in cattle populations, and with no clear disease association. Its genome contains a long unique coding region (LUR) flanked by polyrepetitive DNA and 79 open reading frames (ORFs), with unique 17 ORFs, named Bo1 to Bo17. In 2009, a BoHV-4 strain was isolated (FMV09-1180503: BoHV-4-FMV) from cattle with respiratory disease from Quebec, Canada, and its LUR was sequenced. Despite the overall high similarity, BoHV-4-FMV had the most divergent LUR sequence compared to the two known BoHV-4 reference strain genomes; most of the divergences were in the Bo genes and in the repeat regions. Our phylogenetic analysis based on DNA polymerase and thymidine kinase genes revealed that virus isolate was BoHV-4 gammaherpesvirus and clustered it together with European BoHV-4 strains. Because BoHV-4-FMV was isolated from animals presenting respiratory signs, we have updated the BoHV-4 Canadian cattle seroprevalence data and tried to find out whether there is a link between clinical manifestation and BoHV-4 seropositivity. An indirect immunofluorescence assay (IFA) was performed with nearly 200 randomized sera of dairy cattle from two Canadian provinces, Quebec (*n* = 100) and Ontario (*n* = 91). An additional set of sera obtained from Quebec, from the healthy (*n* = 48) cows or from the animals experiencing respiratory or reproductive problems (*n* = 75), was also analyzed by IFA. BoHV-4 seroprevalence in Canadian dairy cattle was 7.9% (Quebec: 6% and Ontario: 9.9%). Among animals from the Quebec-based farms, diseased animals showed higher BoHV-4 seropositivity than healthy animals (*P* < 0.05), with a significant 2.494 odds ratio of being seropositive in sick compared to healthy animals. Although there is no established direct link between BoHV-4 and specific diseases, these seroprevalence data suggest the possible involvement of BoHV-4 in dairy cattle diseases.

## Introduction

Bovine gammaherpesvirus 4 (BoHV-4) is a widespread cattle virus with no clear association with disease. It has been isolated from animals with a variety of clinical manifestations, yet it also produces a lifelong and asymptomatic infection in the majority of affected animals (1, 2). BoHV-4 has been described mainly as a secondary pathogen implicated in reproductive disorders of cattle (3, 4). However, recently a potential pathogenic role of BoHV-4 in the development of the bovine dermatitis, pyrexia, and hemorrhagic syndrome was suggested (5). It belongs to the *Herpesviridae* family, *Gammaherpesvirinae* subfamily, and *Rhadinovirus* genus (6). This virus shares molecular similarity with human gammaherpesviruses Epstein–Barr virus and *Herpesvirus saimiri* (1, 7). However, BoHV-4 presents distinct antigenic and genomic characteristics compared to bovine alphaherpesvirus 1, 2, and 5 (BoHV-1, BoHV-2, and BoHV-5, respectively), which are placed within the *Alphaherpesvirinae* subfamily (4, 6, 8).

Unlike most other gammaherpesviruses, BoHV-4 is able to easily replicate in a variety of cell lines *in vitro* and in a broad range of host species *in vivo* (7, 9). The natural host of the virus is primarily cattle, but several ruminant and non-ruminant species are susceptible to BoHV-4 infection (10, 11). Wild African buffaloes (*Syncerus caffer*) can be considered as a reservoir species, in which the virus is highly prevalent (11, 12). This virus was first isolated in Europe from cattle with respiratory diseases (13) and later in the United States (14). Since then, more than 40 BoHV-4 strains have been isolated worldwide (11). These genomic and antigenic related strains can be classified into three groups based on genomic analysis: Movar 33/63-like viruses, mostly isolated from Europe; DN599-like viruses, mostly isolated from North America; and African buffalo strains (1, 11–15).

The BoHV-4 genome is a double-stranded DNA of 144 ± 6 kb, composed of a long unique coding region (LUR) containing at least 79 open reading frames, flanked at both ends by multiple copies of tandem repeats, called polyrepetitive DNA (1, 7, 16). The entire genome of two BoHV-4 strains, one from the United States (strain 66-p-347) (7) and the other from Belgium (strain V.test) (17), have been sequenced so far. Although BoHV-4 genome organization features, which are potentially important for viral replication, have already been identified (17), the function of some of the genes remains unknown.

The pathogenic role of BoHV-4 is still not clear, since it was isolated from a wide variety of clinical cases as well as from apparently healthy animals (1, 9, 13). BoHV-4 has been found in cattle affected by respiratory disorders (13), metritis, endometritis, and pelvic inflammatory disease (4, 18–20), orchitis, vulvovaginitis, and abortion (3, 21, 22), neurological disorders (23), diarrhea and skin lesions (1, 24), and rare bovine dermatitis, pyrexia, and hemorrhagic syndrome (5). The viral DNA was also detected in semen (25), in aborted fetuses (26), and milk, and considered as a potential cause of mastitis (27–29). Additionally, the potential of BoHV-4 transmission *via* embryo transfer was recently reported (30). However, several clinical trials of controlled infection failed to reproduce disease, and no correlation was found between the presence of histological lesions and BoHV-4 infection (1, 4, 9).

As for other herpesviruses, BoHV-4 is able to establish latent infection (31). Although the presence of BoHV-4 has been demonstrated in many tissues, one site of persistence in both natural and experimental hosts is the cells of the macrophage/monocyte lineage (31, 32). Other potential sites of viral persistency are the trigeminal ganglia (TG), because it was shown that BoHV-4 DNA was present in the TG of naturally infected cattle, similar to the BoHV-2, which usually establish latency in neuronal tissues (33). Virus reactivation is possible following dexamethasone treatment and stress factors and, frequently, virus reactivation occurs in the absence of clinical disease (32). Thus, the latent character of BoHV-4 is important in the epidemiology and represents a major hindrance to clinical diagnosis.

Currently, it is still uncertain whether BoHV-4 plays a primary role in pathological processes in diseased animals or can potentiate clinical manifestations associated with the infection by another pathogen. Overall, the published data suggest that the pathogenic potential of the virus is low. However, BoHV-4 can exacerbate the clinical impact only when it is present with other infectious pathogens, which may induce reactivation of BoHV-4 from latency (3, 29). BoHV-4 is currently considered as a cofactor for the development of disease usually initiated by bacteria (3, 4, 34). Nevertheless, there is little information about the role of BoHV-4 in cattle diseases and about the clinical significance of coinfection with BoHV-4 and other animal viruses.

In 2009, the nasopharyngeal swabs from two dairy cows with pneumonia were submitted to the Diagnostic Veterinary Virology Laboratory (DVVL) of the Faculté de médecine vétérinaire (FMV) of the Université de Montréal (UdeM). The animals were located in a Quebec (Canada) dairy farm experiencing an outbreak of respiratory and reproductive diseases. The farm had 200 Holstein cows housed in free stalls. Several multiparous periparturient cows (*n* = 22) presented at least one of the following clinical conditions between April and May 2009: acute mastitis (*n* = 3), milk fever (*n* = 8), ketosis (*n* = 6), displaced abomasum (*n* = 1), downer cow (*n* = 1), retained placenta (*n* = 3), birth of stillborn calves (*n* = 3), abortion (*n* = 1), or pneumonia (*n* = 3), and three of those cows died. In June, a total of six cows died, and there were seven cows with pneumonia. Of those cows, four did not calved as expected during the summer period. Two tracheal aspirates were collected from two different cows experiencing pneumonia. Bacteria isolation diagnostic assays in one sample revealed the presence of *Mycoplasma bovis* and, in the second sample, revealed the presence of *Arcanobacterium pyogenes, Pasteurella multocida*, and *Histophilus somni*. This dairy herd was vaccinated on a continuous basis against BoHV-1, bovine viral diarrhea virus, parainfluenzavirus 3, and bovine respiratory syncytial virus in the first 30 days after calving, and against *Escherichia coli* mastitis during the dry period. Importantly, the BoHV-4 was isolated in cell culture from the two submitted swab samples. This intriguing finding prompted us to further characterize the virus genome using the next-generation sequencing (NGS) technology in attempt to determine whether the new BoHV-4 isolate was different from the known 66-p-347 and V.test BoHV-4 reference strains. Since very limited epidemiological data are available on the prevalence of BoHV-4 in Canada, a diagnostic serological assay was developed and used to investigate the prevalence of the BoHV-4 in Canadian healthy and sick dairy animals and to establish if any correlation exists between BoHV-4 and cattle diseases.

## Materials and Methods

### Cells

Madin–Darby bovine kidney cells (MDBK, ATCC CCL-22, American Type Culture Collection, VA, USA) were used for BoHV-4 isolation and for serological assays, i.e., the immunofluorescence assay (IFA). The MDBK cells were cultured in minimum essential medium with Earle’s salts (MEM) (Invitrogen Corporation, GibcoBRL, Burlington, ON, Canada), supplemented with 10% fetal bovine serum (FBS) (Wisent Inc., St-Bruno, QC, Canada), 300 U/mL of penicillin, 300 µg/mL of streptomycin, 10 mM HEPES (Invitrogen Corporation, GibcoBRL, Burlington, ON, Canada), and maintained at 37°C and 5% CO_2_ in a humidified incubator.

### Virus Isolation

The nasopharyngeal swabs from two dairy cows with pneumonia were submitted to the DVVL of the Faculté de médecine vétérinaire (FMV) of the UdeM, in 2009. The animals were located in a Québec (Canada) dairy farm experiencing an outbreak of respiratory and reproductive diseases. Nasopharyngeal swab samples were immersed in MEM containing antibiotics at a concentration that was three times higher than described for maintenance of cell cultures, and were vigorously shaken and centrifuged at 750 *g* for 10 min. The supernatants were filtrated (0.45 µm filter) before inoculation of 80% confluent monolayers of MDBK cells. Infected cells were incubated in MEM supplemented with 2% FBS at 37°C and 5% CO_2_ for 4 days. Virus isolation was conducted during three consecutive passages. The cultures were examined daily for the presence of cytopathic effect (CPE). Intracellular virions were released by three cycles of freezing at −80°C and thawing. Cellular debris was removed by centrifugation at 750 *g* for 10 min and the supernatant containing virions were stored at −80°C. Non-inoculated MDBK cells served as a negative control. To confirm the presence of the virus isolate (named FMV09-1180503/BoHV-4-FMV), a transmission electron microscopy (TEM), a pan-herpesvirus nested PCR (nPCR) assay, and a specific IFA were conducted on virus-infected cell culture and cell lysates as described below.

### Transmission Electron Microscopy

When a CPE in infected cell culture was observed, the cell culture supernatant was first concentrated and then purified on a sucrose gradient by ultracentrifugation. Purified viruses were fixed with a 2% glutaraldehyde solution, stained and examined by TEM (Philips, model EM201 and Hitachi, model HT7700, Rexdale, ON, Canada). Briefly, 50 µL of purified-fixed viruses were ultracentrifuged (Airfuge, Beckman Coulter, Inc., Mississauga, ON, Canada) at 100,000 *g*/30 min to pellet virions directly onto a formvar coated copper grid (Canemco & Marivac, QC, Canada). The grids were then negatively stained using 2% phosphotungstic acid and 2% uranyl acetate and visualized by TEM for the detection of viral particles.

### Pan-Herpesvirus nPCR Assay

Briefly, the pan-herpesvirus nPCR assay was performed as previously described (35). The nPCR amplified a variable region of the viral DNA polymerase (DPOL) gene. Thereafter, the PCR product, of more than 200 nucleotides in length, was sequenced (Sequencing Laboratory, FMV, Université de Montréal, QC, Canada) to confirm the presence of herpesvirus sequences.

### Immunofluorescence Assay

For the identification of BoHV-4, monolayers of MDBK infected cells were fixed with cold 80% acetone solution for 10 min when CPE was evident (2 days after infection). After fixation, cells were rehydrated in phosphate-buffered saline solution (PBS, pH 7.2) and incubated for 30 min in a 1/30 dilution of rabbit anti-BoHV-4 serum (courtesy provided by Dr. Fernando Osorio, University of Nebraska, Lincoln, NE, USA). Subsequently, cell monolayers were washed three times with PBS and incubated for 30 min in a 1/40 dilution of fluorescein isothiocyanate (FITC) conjugated goat anti-rabbit serum (Zymed Laboratories, San Francisco, CA, USA) and then washed three times with PBS. Cells were visualized using a DMI 4000B reverse fluorescence microscope and photographic images were taken with a DFC 490 digital camera (Leica Microsystems Inc., Richmond Hill, ON, Canada).

To evaluate the seroprevalence of BoHV-4 and BoHV-1 in Quebec and Ontario cattle, antibodies titers were determined with an IFA (36). The virus strains used were FMV09-1180503 (BoHV-4-FMV) and ATCCVR-864 (BoHV-1). Briefly, MDBK cells growing in 96-well microplates were infected with BoHV-1 or -4. Infected cells were incubated in MEM supplemented with 2% FBS at 37°C and 5% CO_2_. When CPE became apparent (2 days post-infection), infected cells were fixed with 4% paraformaldehyde in PBS solution. Cell monolayers were permeabilized by adding 0.1% Triton X-100 solution (in PBS) during 10 min at room temperature. After the permeabilization procedure, cells were incubated 30 min in PBS containing 0.2% Tween 20 and 1% FBS. Cows’ sera were diluted through a series of twofold dilutions from 1/4 to 1/512 in the washing buffer (0.02% Tween 20 in PBS) in separate microplates, added to the infected cells and incubated at room temperature for 90 min. Then, cell monolayers were washed three times with a solution containing 1% FBS in PBS-Tween 20 and incubated for 60 min in the washing buffer containing a 1/100 dilution of rabbit anti-bovine FITC conjugate antibody (5 mg/mL, MP Biomedicals, Solon, OH, USA). Finally, cell monolayers were washed three times as described above and observed by the DMI 4000B reverse fluorescence microscope. Mock-infected cells and BoHV-1 and -4 in-house selected positive bovine sera were used as controls. At the time when this study was conducted, the commercially available ELISA for BoHV-4 was not widely available. Since the manufacturing company is located in Belgium, we assumed that commercially available kit (BioX Diagnostics) was designed for European BoHV-4 strain. Therefore, it was crucial for our project to develop a serological assay using BoHV-4 Canadian field-related viral antigens such as the BoHV-4 isolated strain. Consequently, an IFA assay was developed using BoHV-4-FMV strain.

### High Throughput Sequencing

Bovine gammaherpesvirus 4-FMV-infected MDBK cells were frozen and thawed three times. The lysates were precleared by centrifugation at 3,000 *g* for 20 min and supernatants were subjected to ultracentrifugation through 30% sucrose cushion in TNE buffer (50 mM Tris, pH 7.5; 140 mM NaCl and 5 mM EDTA), at 112,000 *g* for 5 h, using the SW28 Beckman Coulter rotor (Beckman Coulter Canada Inc., Mississauga, ON, Canada). The pelleted virus was resuspended in TNE buffer and ultracentrifuged through a cesium chloride (CsCl) equilibrium gradient (in TNE buffer) for 6 h at 154,000 *g*, using Sorvall TH-641 swinging bucket rotor (Kendro Laboratory Products, Newtown, CT, USA). A visible whitish band was collected by aspiration and the presence of the viral particles was confirmed by the pan-herpesvirus nPCR assay. CsCl purified virions were resuspended in 2 mL TE solution (10 mM Tris–hydrogen chloride [HCl], pH 7.5, 1 mM EDTA) containing 0.5% sodium dodecyl sulfate and 100 µg/mL of proteinase K, which was mixed by brief agitation, then heated at 55°C for 30 min. Viral DNA was extracted by phenol-chloroform-isoamylalcohol (25:24:1) and thereafter precipitated with ethanol and stored at −20°C. The purified viral DNA was submitted to the McGill University Génome Québec Innovation Center sequencing laboratories. The sample was sequenced on a Roche 454 GS FLX instrument using the Titanium chemistry. A total of 82,194 reads for a total of 23,875,823 bases was obtained which corresponds to a coverage of more than 200-folds of the LUR region of the viral genome. Genome assembly was performed using Newbler software v2.6 (Roche, Life Technology). Sequence finishing was done using CLC Genomic Workbench v5 (CLC Bio, Redwood City, CA, USA). Gaps in the sequence assembly were resolved by performing PCR amplification covering these gaps and sequencing the amplicons by the Sanger method.

### DNA Sequence Analysis

A pairwise comparison between the three complete BoHV-4 genomes available in GenBank (accession numbers KC999113, AF318573, and JN133502 for BoHV-4-FMV, 66-p-347, and V.test, respectively) was performed. The number of substitutions and gaps between sequences was recorded in one-nucleotide sliding windows of 501 bp using the Python script.

The nucleotide sequences of the DPOL and the thymidine kinase (TK) genes were used to construct the molecular phylogenetic trees for different strains of herpesviruses. A set of herpesvirus sequences was available from GenBank sequences (non-redundant NT) with Blast+ (37). The sequences of the DPOL and the TK genes of BoHV-4-FMV were used as the query sequence. DNA sequences were then translated with transeq (38), and the multiple protein sequence alignment was performed with Kalign2 (39). The alignment was manually validated and corrected with SeaView (40). This allowed to align the nucleotide sequences with TranAlign software (38). Nucleotide sites for the phylogeny were selected with Gblocks (41). For the DPOL set, a total of 40 sequences (Table S1 in Supplementary Material) were used for herpesviruses (*Alphaherpesvirinae, Betaherpesvirinae*, and *Gammaherpesvirinae*) molecular phylogeny with a length of 1,555 nucleotides. For the TK set, a total of 27 sequences (Table S2 in Supplementary Material) were used for BoHV-4 strains phylogeny with a length of 212 nucleotides.

Molecular phylogenetic trees were inferred from DNA sequences with the MrBayes program (42). The general time reversible model of nucleotide evolution with 16 Markov chain Monte Carlo analyses were applied over 4 runs for 400,000 generations, sampling every 100 generations. Branch lengths are proportional to genetic distances (the scale bar units are substitutions per aligned position).

### Epidemiological Survey

A first set of serum samples analyzed in this study was convenient random samples collected in 2009 from dairy cattle herds that were submitted to two diagnostic laboratories for different purposes (such as health surveillance and vaccination status). One sample was randomly collected from each herd. A total of 191 sera were obtained from DVVL (Faculté de médecine vétérinaire, Université de Montréal, Québec, QC, Canada) and Animal Health Laboratory (University of Guelph, ON, Canada) for a general epidemiological assessment of BoHV-4 seroprevalence.

A second set of serum samples (*n* = 123) was collected in 2009 and 2010 from specifically selected Quebec dairy cattle herds with no health issue (*n* = 48) or documented clinical problems (*n* = 75). The serum samples from healthy and clinically affected herds were randomly obtained from 4 herds (5–15 samples/herd; one sample/animal) and 12 herds (1–15 samples/herd; one sample/animal), respectively. From the clinically affected herds, only diseased animals were sampled. Several animals from diseased herds were experiencing respiratory problems, cough, calving problems, abortion, embryonic death, retained placenta, metritis, mastitis, and unexplained fever that were affecting the herd performance. The BoHV-4 seroprevalence of healthy and diseased animals was determined by IFA and results obtained from both populations were compared.

### Statistical Analyses

The serological data were not normally distributed. Thus, non-parametric analyzes were conducted using the Mann–Whitney unpaired test. A contingency table with the Fisher’s exact test was used to determine whether the prevalence of BoHV-4 was significantly higher in diseased dairy cattle compared to healthy animals. The GraphPad Prism software version 5.03 (La Jolla, CA, USA) was used for the statistical analyzes. Values of *P* < 0.05 were considered to be significant.

## Results

### BoHV-4 Isolation and Identification

**Figure 1:**
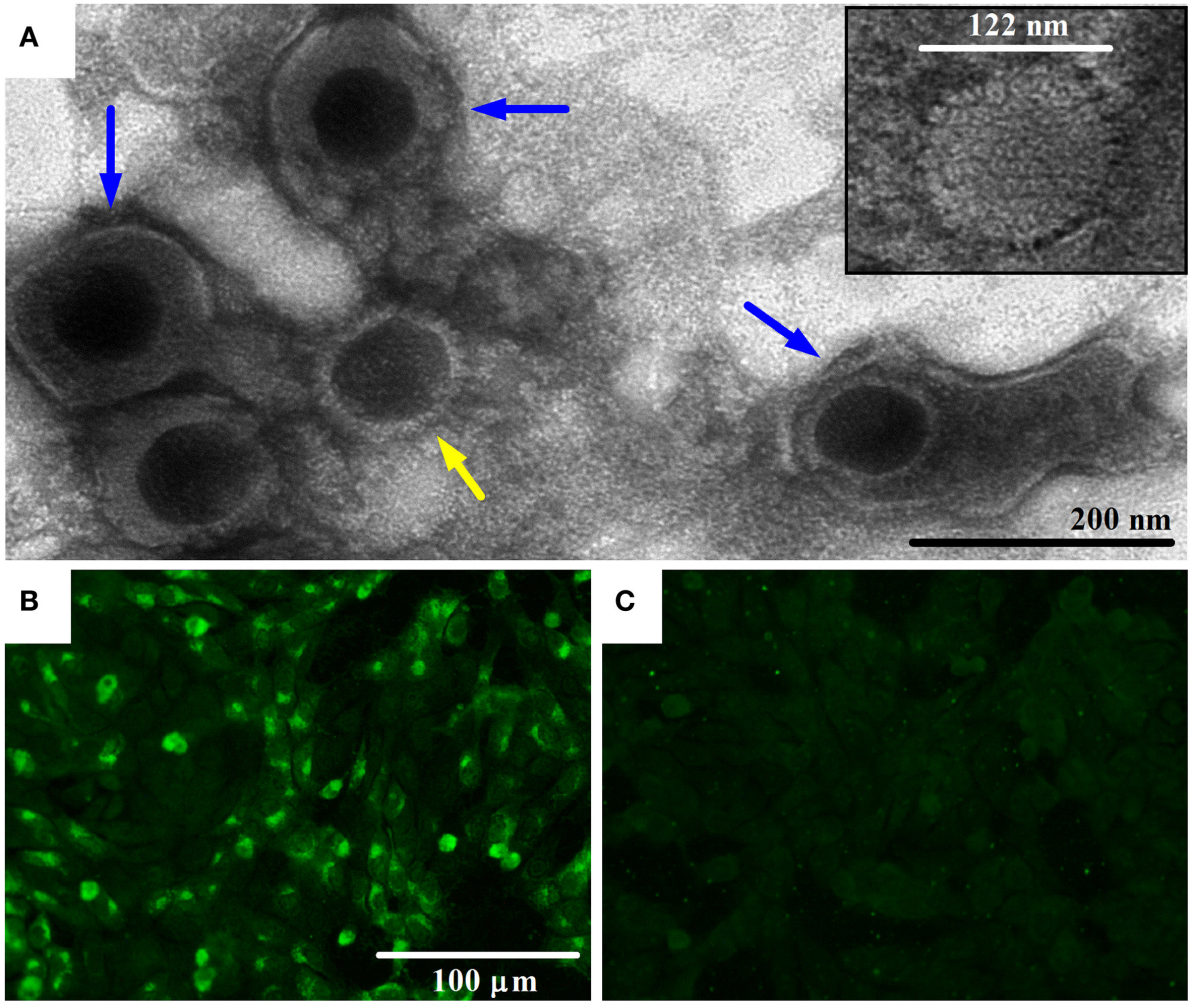
Transmission electron microscopy (TEM) image of bovine gammaherpesvirus 4 (BoHV-4)-FMV isolate and detection of its antigens by indirect immunofluorescence assay (IFA). **(A)** TEM image of BoHV-4-FMV virus isolate grown on MDBK cells, showing herpesvirus-like virions. Negative-stain TEM allows the visualization of enveloped (blue arrows) and non-enveloped (yellow arrow) herpesvirus particles. A more detailed icosahedral capsid structure of the virus with an overall size of 122 nm in diameter, with its capsomere subunits, is illustrated in the inset. **(B)** Visualization of BoHV-4-FMV MDBK-infected cells by a specific IFA. **(C)** Mock MDBK-infected cells.

Bovine gammaherpesvirus 4 was isolated from cattle experiencing respiratory problems by infecting MDBK cells. The CPE observed was rounding cells in a shape of grape clusters, cellular death, and monolayer detachment (data not shown). The herpesvirus presence was confirmed by the visualization of the icosahedral viral capsid (measuring around 100–125 nm in diameter) and enveloped virus particles morphologically similar to viruses of the *Herpesviridae* family using negative-stain electron microscopy (Figure 1A), and by pan-herpesvirus nPCR assay (35) (data not shown). The sequencing of the pan-herpesvirus nPCR amplicon was done to confirm the identity of the virus. Immunofluorescent staining with a specific antibody against BoHV-4 revealed the presence of viral proteins in the nucleus and cytoplasm indicating the presence of the virus in infected cells (Figure 1B).

### BoHV-4 Sequence Analysis

**Figure 2:**
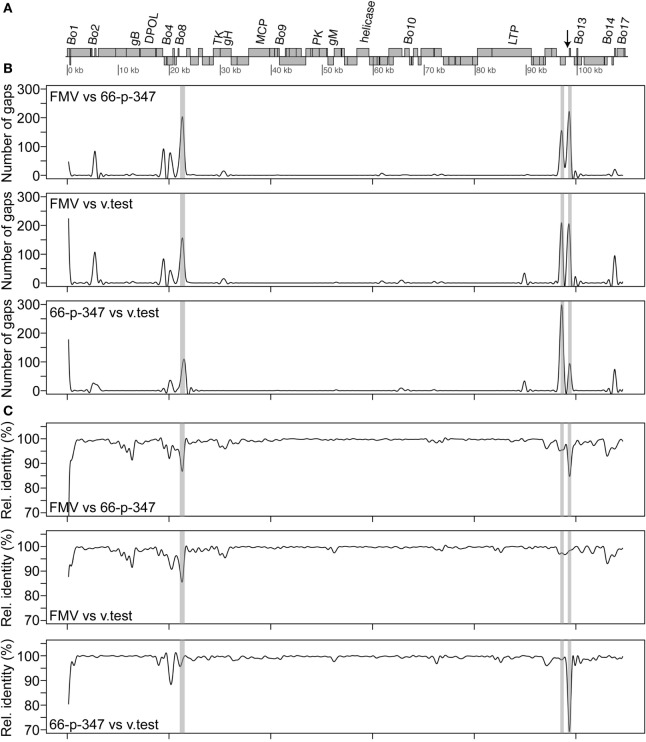
Sequence comparison of the bovine gammaherpesvirus 4 (BoHV-4) long unique coding region (LUR) sequences. **(A)** Map of the BoHV-4 LUR genome. Every open reading frame is illustrated by a gray box. Upper boxes correspond to genes on the + strand, while the others are the genes on the − strand. Only some specific genes are indicated. The arrow indicates the position of the quasi-palindromic motif of the oriLyt region. **(B)** A pairwise comparison of the number of gaps between the three complete BoHV-4 genome sequences [FMV (BoHV-4-FMV), 66-p-347, and V.test] is shown. **(C)** A pairwise comparison of the relative identity between the three complete BoHV-4 genome sequences is shown. For both B and C, a 501 bp sliding window was used for the comparison. The gray areas shown in B and C represent the repeat regions (R1, R2a, R2b from left to right) found in the BoHV-4 genomes.

Sequence assembly of reads from Roche 454 GS FLX and amplicon sequencing produced a sequence of 108,349 bp in length. This sequence corresponds to the LUR of BoHV-4-FMV (Figure 2A) and was submitted to GenBank (accession number KC999113). Overall, the genome of BoHV-4-FMV is quite similar to the two other LUR BoHV-4 genomes already published for 66-p-347 and V.test reference strains (7, 17).

Detailed analysis revealed that punctual high levels of variability were present in various regions of the LURs (Figures 2B,C). Pairwise comparisons showed that regions with higher variability were mostly the same in the three virus sequences. Gaps were clearly more frequently located within the repeat regions than elsewhere in the genomes (Figure 2B). Important variations of the relative identity between the sequences were also seen within the repeat regions but also elsewhere (Figure 2C). Outside the repeat regions, sequence variability between the BoHV-4 sequences was mostly observed within or in the vicinity of some Bo genes (Bo1, Bo3, Bo5, Bo8, Bo11, Bo12, Bo16, and Bo17). Based on the profiles shown in Figure 2 and particularly those of relative identity, it is tempting to propose that BoHV-4-FMV is the most divergent of the three strains. In order to confirm that, global distances between genome sequences were calculated using Kimura 2 parameters (K2p) substitution model. All gap positions were excluded before computing the values. The distances (in numbers of substitution per site) between genomes are as follows: 0.0142 (BoHV-4-FMV versus 66-p-347), 0.0122 (BoHV-4-FMV versus V.test) and 0.0093 (66-p-347 versus V.test). The higher value of distance indicates a greater level of divergence between strains. Therefore, these results demonstrated that BoHV-4-FMV strain has the most diverging LUR sequence from the known BoHV-4 genomes.

**Figure 3:**
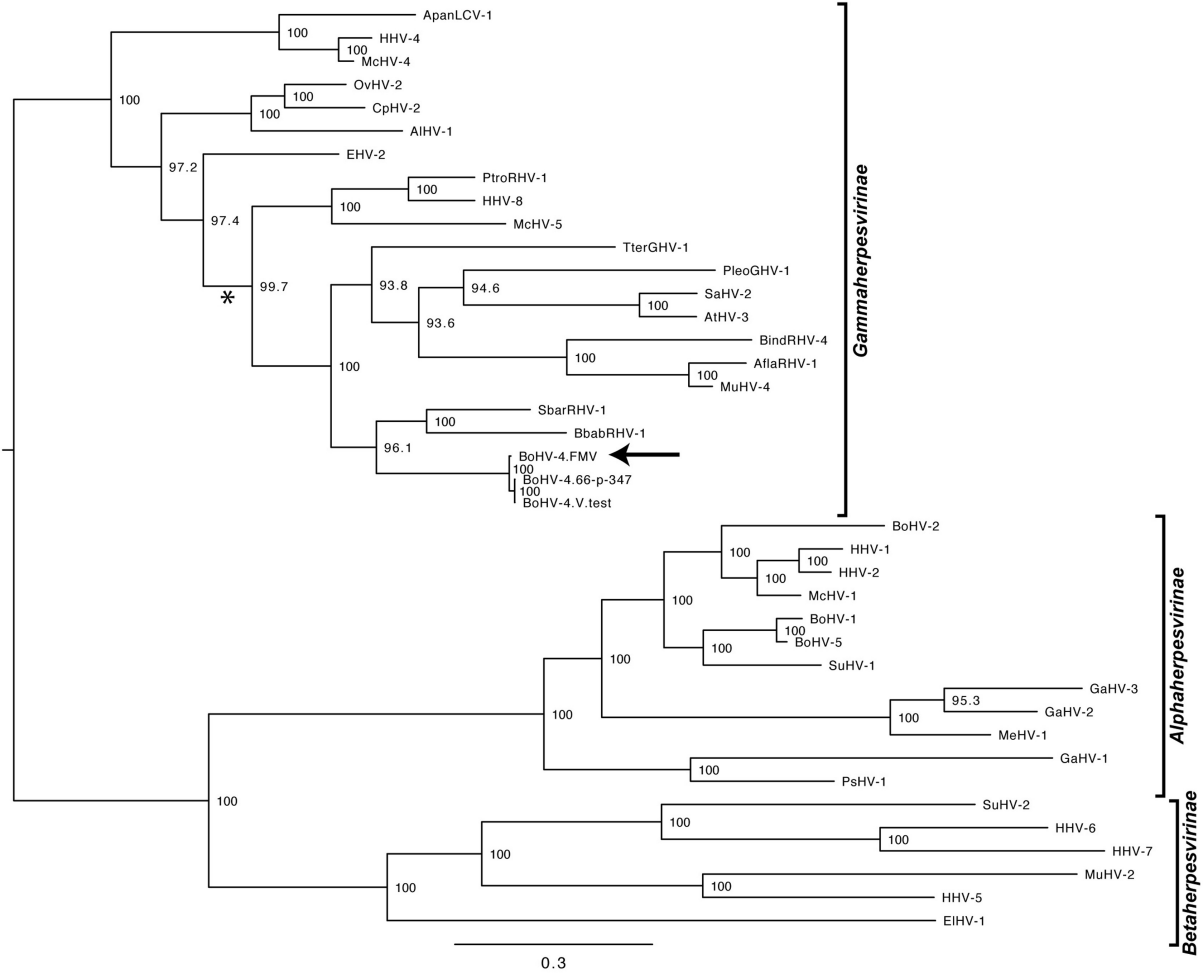
Molecular phylogeny of the DNA polymerase (DPOL) gene of various herpesvirus species. The phylogeny of DPOL gene sequences (1555 nucleotides in length) from 40 herpesviruses (Table S1 in Supplementary Material) was determined using MrBayes programs. Numbers on branches correspond to posterior probabilities. Branch length is proportional to the number of nucleotides substitutions. The tree is unrooted. The arrow points to the bovine gammaherpesvirus 4 (BoHV-4)-FMV strain and the asterisk (*) highlights the *Rhadinovirus* genus cluster.

As it was expected, the molecular phylogenetic analysis using the DPOL gene sequence of 40 herpesvirus species positioned the BoHV-4-FMV as well as the other BoHV-4 strains within the *Gammaherpesvirinae* subfamily (Figure 3) (7). More particularly, it appeared that BoHV-4 viruses grouped with herpesviruses infecting babirusa and Bornean bearded pigs. In the *Gammaherpesvirinae* subfamily, all the viruses within *Rhadinovirus* genera are clustered together (see * in Figure 3), which includes the BoHV-4 viruses as well as two viruses with unassigned genus (TterGHV-1 and PleoGHV-1). Based on the phylogenetic tree, it is possible that the genus of these two viruses could also be *Rhadinovirus*.

**Figure 4:**
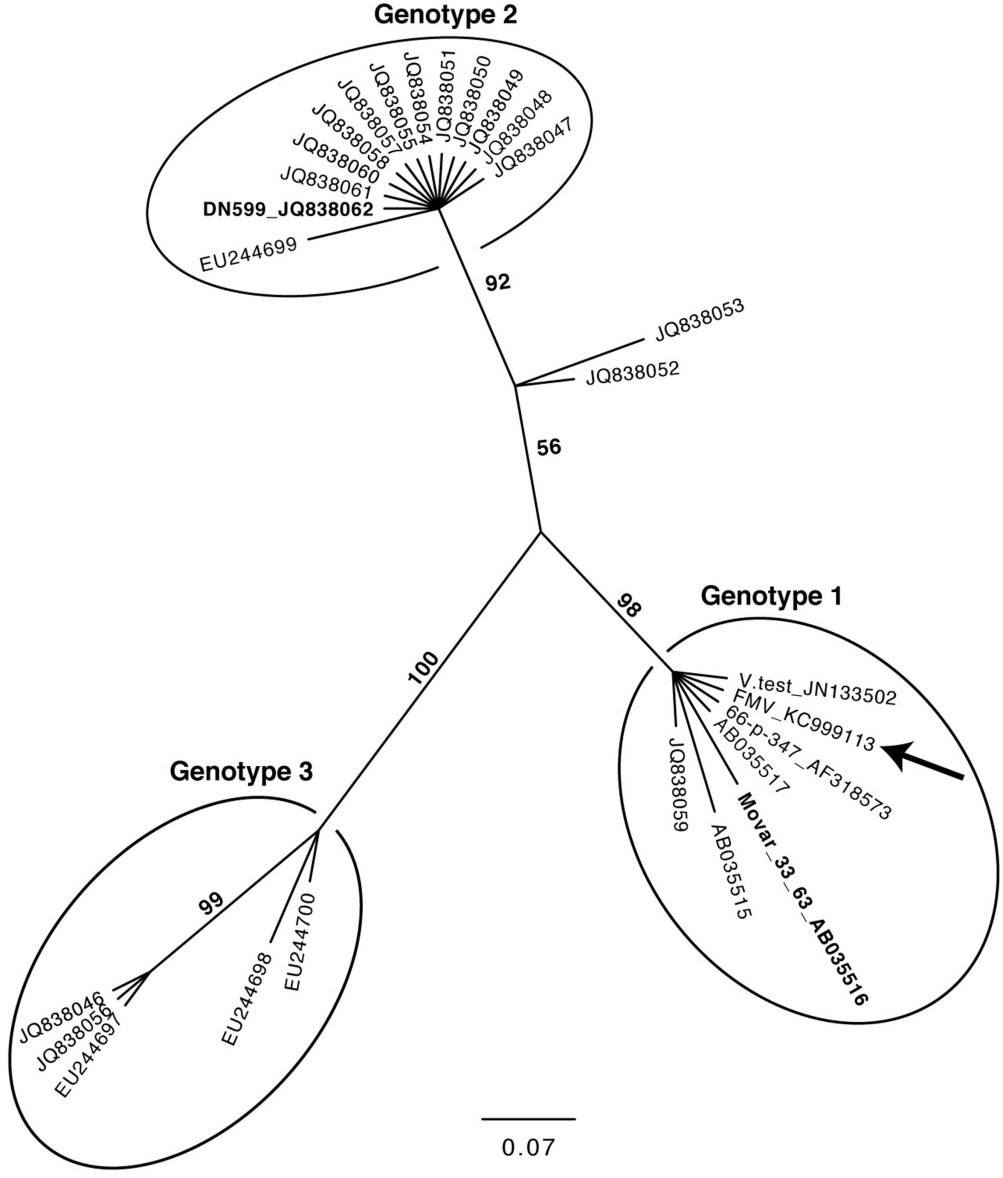
Molecular phylogeny of the Thymidine kinase (TK) gene from various bovine herpesvirus 4 strains. The phylogeny of TK gene sequences (212 nucleotides in length) from 27 bovine gammaherpesvirus 4 (BoHV-4) strains (Table S2 in Supplementary Material) was determined using MrBayes programs. Numbers on branches correspond to posterior probabilities. Branch length is proportional to the number of nucleotides substitutions. The tree is unrooted. The arrow points to the BoHV-4-FMV strain. The reference strains Movar_33_63 and DN599 are in bold.

The molecular phylogeny of 27 BoHV-4 sequence strains obtained, using the TK gene is shown in Figure 4. This phylogenetic tree was constructed in a similar way as it was described by Verna et al. (43), using the same dataset and adding the sequence of the TK gene of the three BoHV-4 strains for which a complete genome is available (BoHV-4-FMV, V.test, and 66-p-347). The very same phylogeny was reproduced, while demonstrating that the TK gene of the three additional strains (BoHV-4-FMV, V.test, and 66-p-347) was clustered within the Genotype 1 group.

### BoHV-4 Seroprevalence in Canada

A low BoHV-4 seroprevalence was observed in cattle from Quebec and Ontario (6 and 9.9%, respectively). In contrast, the seroprevalence of BoHV-1 was much higher and reached 38% in Quebec and 37.4% in Ontario (Table 1). The relatively high BoHV-1 seroprevalence was probably due, in part, by the broad use of BoHV-1 commercial vaccine in dairy cattle herds (8, 44). The seroprevalence of BoHV-4 and BoHV-1 for both provinces taken together were 7.9 and 37.7%, respectively. The cattle of both provinces seem to share similar serological patterns, since the differences between their respective BoHV-4 and BoHV-1 seroprevalence were not significant (Table 1). The seroprevalence of BoHV-1 was used as a control in our study. Moreover, it was decided to test for BoHV-1 seroprevalence in order to evaluate the possible cross-reactivity of the BoHV-4 antibodies. Interestingly, several animals, which were negative for BoHV-1, were positive for BoHV-4, and *vice versa*, indicating that serological cross-reaction between BoHV-1 and BoHV-4 antibodies was not a major concern to the present study.

**Table 1:** Bovine gammaherpesvirus 4 (BoHV-4) and 1 (BoHV-1) seroprevalence in Quebec and Ontario provinces.

BoHV-4 seroprevalence			BoHV-1 seroprevalence		
Quebec samples (*n* = 100)	Ontario samples (*n* = 91)	Overall (*n* = 191)	Quebec samples (*n* = 100)	Ontario samples (*n* = 91)	Overall (*n* = 191)
6.0%	9.9%	7.9%	38.0%	37.4%	37.7%

*No significant statistical difference (*P* > 0.05) was observed between Quebec and Ontario seroprevalence for both BoHV-4 and BoHV-1 using the non-parametric Mann–Whitney test*.

Serological analyses of the second set of samples (from healthy and diseased animals from Quebec) revealed that BoHV-4 seroprevalence was higher in animals presenting clinical signs of diseases (respiratory/reproductive problems) compared to healthy animals (50.6% compared to 29.2%, respectively) (*P* < 0.05) (Table 2). Thus, clinically affected animals have a significantly higher risk to be seropositive for BoHV-4 compared to healthy animals (with a 2.494 odds ratio; Table 2). On the other hand, no significant difference in BoHV-1 seroprevalence was observed between healthy and diseased dairy cattle (60.5% compared to 60%, respectively) (Table 2). Interestingly, a tendency toward higher antibody titers against BoHV-4 was observed in diseased animals compared to healthy animals (29.3 and 14.6% of animals with titers between 128 and 512, respectively). No difference was observed in the prevalence of animals having different levels of BoHV-1 antibody titers between diseased and healthy dairy cattle (Table 2).

**Table 2:** Bovine gammaherpesvirus 4 (BoHV-4) and 1 (BoHV-1) seroprevalence in diseased dairy cattle.

		BoHV-4 seroprevalence		BoHV-1 seroprevalence	
Immunofluorescence assay results	Antibodies titers	Healthy animals %/(*n*)	Diseased animals %/(*n*)	Healthy animals %/(*n*)	Diseased animals %/(*n*)
Negative	<8	70.8/(34)	49.3/(37)	39.6/(19)	40.0/(30)
Positive	8–64	14.6/(7)	21.3/(16)	43.8/(21)	41.3/(31)
	128–512	14.6/(7)	29.3/(22)	16.7/(8)	18.7/(14)

	Total	100 (48)	100 (75)	100 (48)	100 (75)
		OR^a^: 2.494 [1.155–5.386]*		OR: 0.983 [0.4687–2.061]	

*^a^OR, odds ratio. Numbers in brackets represent the 95% confidence interval*.

*P < 0.05 (Fisher’s exact test).

## Discussion

The isolation of BoHV-4 from dairy cattle experiencing respiratory problems was an unexpected finding because this virus has never been isolated from cell cultures for the past 12 years in our diagnostic laboratory (DVVL). However, a diagnostic assay for bacteria isolation revealed the presence of *M. bovis* in one sample. In the second swab sample, the assay revealed the presence of *A. pyogenes, P. multocida*, and *H. somni* (data not shown). Due to the identification of *M. bovis* (and other bacteria) in the same sample, BoHV-4 was not considered to be the primary etiological agent of the respiratory disease. Unfortunately, no data on *M. bovis* seroprevalence are available for the Quebec’s herds included in the present study. In a recent study realized in Quebec, *M. bovis* has been detected by PCR and culture in nasal swabs of 20% of dairy calves, in a relatively small size sample (45). Furthermore, it was found that the prepartum nutritional program of the herd was deficient in vitamin E, energy, and protein (data not shown), which also could contributed to the health status of the dairy cattle. Nonetheless, since very limited data about the BoHV-4 prevalence, the type of circulating viral strains, and their pathogenic potential in Canada are available, we aimed to investigate these research questions.

The complete viral LUR sequence of the Canadian BoHV-4 isolate (FMV09-1180503, BoHV-4-FMV) was sequenced with the help of deep sequencing technology. Next, the analysis of the LUR sequence of BoHV-4-FMV strain was compared with the two other available BoHV-4 LUR viral genomes. It appeared that the BoHV-4-FMV strain was the most divergent viral genome in this group. Notably, virus genomic differences can potentially lead to different pathogenic potential. This was recently demonstrated *in vitro* when replication kinetics and virus titers of different BoHV-4 strains were compared (46). Most of the divergences between BoHV-4 genomes were in the Bo genes and in the repeat regions (Figure 2). In fact, these regions are in general those with the highest divergence between the three BoHV-4 strains.

As expected, the DPOL gene phylogenetic analysis confirmed that BoHV-4-FMV belongs to *Gammaherpesvirinae* (Figure 3) (7). However, it was surprising that the phylogeny of the TK gene placed the BoHV-4-FMV Canadian isolate within the European group (Genotype 1) (43). The V.test and 66-p-347 strains also belong to this genotype, which seems to be logical because both have been isolated in European countries. There are three possible explanations for the unexpected grouping of the BoHV-4-FMV strain within genotype 1. First possibility: this strain is originally from Europe and was introduced into North America by the importation of animal carriers or contaminated bovine products. Second, as it was suggested by Verna et al. (43), the phylogenetic analysis based only on the TK gene is not powerful enough, and additional markers or strains are needed in order to create a more accurate picture of the BoHV-4 genomic diversity. Third, the unexpected grouping is a result of previous recombination events. In fact, evolution of herpesviruses for a long time has been attributed, at least in part, to the process of recombination. The NGS technologies have greatly increased the accuracy of the detection of genetic recombination events, single nucleotide polymorphisms, and minor mutations. Significantly, high rates of *in vivo* intra-species homologous recombinations have been demonstrated, for example, for herpes simplex virus-1 and BoHV-1, which corroborate the important role of recombination in herpesvirus diversification and evolution (47, 48).

Our second goal was to elucidate any potential association between BoHV-4 infection and diseases in cattle. Previous studies showed that the IFA is highly specific for detection of BoHV-4 antibodies and proved that there is no cross-reactivity between BoHV-1 and BoHV-4 (49). Therefore, we used this approach in our study. The present epidemiological survey, conducted in Canadian dairy cattle, showed that BoHV-4 seroprevalence was very low compared to other reports, which may explain why BoHV-4 is rarely detected by Canadian veterinary diagnostic laboratories, including our diagnostic laboratory (DVVL). For instance: compared with the value of 8.9% reported in Japan (50); 23.3% (dairy cattle) to 33.3% (beef cattle) in Northern Ireland (51); 84, 79, and 13.8% in 3 Dutch dairy herds (52); and 36, 83, and 84.4% in animals with a history of metritis from Georgia, USA (53), Spain (54), and Belgrado, Serbia (55), respectively. Other studies that investigated the role of this virus in clinical and/or subclinical mastitis in Canadian dairy cows detected antibodies against BoHV-4 in a high amount of milk samples (98%) (29). It is very hard to reconcile these data with our BoHV-4 seroprevalence results that are much lower. In fact, some details of the experimental design (e.g., the selection criteria of milk samples) are missing in that report, which do not allow us to understand this discrepancy. Notably, it is evident that the subset of milk samples tested by Ali and collaborators was not a random representation of dairy cows (29). In fact, the prevalence of milk samples positive for *Staphylococcus aureus* was 28.5% according to Ali et al. (29), while it was reported to be around 3% for the repository of the Canadian milk samples collected randomly (56). The samples studied by Ali et al. have been taken from this repository. Consequently, we can speculate that discrepancy between these two reports and the discrepancy between Ali et al. and ours data (regarding the BoHV-4 seroprevalence) could be explained by a bias sampling by Ali et al. (29).

More interestingly, the antibody titers against BoHV-4 in the present study were higher in diseased animals compared to healthy animals. Our results suggest that BoHV-4 can potentially have a role in bovine diseases, i.e., can affect the respiratory and reproductive systems of animals. The possibility that BoHV-4 may play a causal role in the development of disease is still uncertain, because the virus has been found in both clinical cases (4, 18, 21, 22, 25, 28) and healthy asymptomatic animals. Nevertheless, an increasing number of studies consider BoHV-4 as a risk factor for reproductive tract infections (21, 22, 31). Several other studies have proposed that BoHV-4 was a secondary agent, which was often associated with concomitant or secondary bacterial infections (3, 4, 34). Therefore, in the context of coinfection and synergy with other pathogens, the higher BoHV-4 seroprevalence in cattle with respiratory or reproductive diseases can be expected. Hence, our study supports the suggestion that BoHV-4 is a frequent risk or a secondary factor in cattle infections/diseases.

The indirect role of BoHV-4 as a potential causative agent of disease in cattle was reinforced by our finding of the concomitant infection by *M. bovis*, which is a well-known pathogen that causes respiratory diseases in cattle (57). The emergence of BoHV-4 can be explained by the presence of a primary agent that triggers the reactivation of latent BoHV-4 infection (3, 29). In fact, it has been demonstrated that BoHV-4 infects macrophages and that immune cells can become persistently infected (31). On the other hand, it has been suggested that BoHV-4 can contribute to disease by lytic replication in host cells such as macrophages, thereby increasing inflammation and contributing to the vascular tissue damage (19, 58). This hypothesis is supported by a positive association between the presence of antibodies against BoHV-4 in cattle and the incidence of bovine mastitis caused by *S. aureus*, suggesting that previous infection with BoHV-4 promotes the development of mastitis caused by bacteria (52). A possible immunosuppressive effect of BoHV-4 on the host immune system has also been studied because persistence and isolation from cells of the immune system (spleen, blood leukocytes) indicate a potential relation to the immune response (9).

Additionally, BoHV-1 seroprevalence was found to be relatively high in our study (37.7% in the overall population and about 60% in diseased animals). However, these data and association with cattle diseases should be investigated further and considered with caution because BoHV-1 vaccines are widely used in Canada.

To our knowledge, this is the first report of the nearly complete viral genome of a Canadian BoHV-4 isolate. When compared against the two reference strains, this Canadian BoHV-4 strain showed a certain level of divergence. In addition, a low BoHV-4 seroprevalence was found in two Canadian provinces. The highest seroprevalence of BoHV-4 has been found in cattle with respiratory and reproductive diseases. However, based on previous reports, it is uncertain whether the BoHV-4 is the primary causative agent of the disease. Thus, it is plausible to suggest that BoHV-4 can act as a synergistic cofactor for some of these diseases, acting as a secondary pathogen that can be triggered in a suitable environment. For example, the immunosuppressive circumstances generated by other pathogens may favor the BoHV-4 reactivation, which consequently will contribute to aggravating any infectious diseases.

## Author Contributions

CB, JL, and SJC carried out the sequencing of viral genome and sequence analyses; LD, NT, and SC selected and provided the clinical samples; NM and J-PA carried out the isolation of virus and serological tests; SM performed the statistical analysis; CP performed electron microscopy research; CT, NM, LA, and CG drafted and edited the manuscript. CG is the scientific director of DVVL and he led and coordinated the project. All authors have read and approved the manuscript as submitted.

## Conflict of Interest Statement

The authors of the present work declare that the research was conducted in the absence of any commercial, financial, or other relationships that could be construed as a potential conflict of interest.
