# Supplementary material for: Whole Genome Sequencing of a Canadian Bovine Gammaherpesvirus 4 Strain and the Possible Link between the Viral Infection and Respiratory and Reproductive Clinical Manifestations in Dairy Cattle
Source: Front Vet Sci. 2017 Jun 16;4:92. doi: 10.3389/fvets.2017.00092 (PMC5472674; doi:10.3389/fvets.2017.00092)
Supplement: Supplementary file 1 [file Table_1.DOCX]

**Supplemental Table 1.**  List of the 40 sequences used in the DPOL gene molecular phylogeny of herpesvirus.

| ***Subfamily: Gammaherpesvirinae*** | ***Genus*** | **Abbreviation** | **Accession** |
| --- | --- | --- | --- |
| *Bovine herpesvirus 4* | *Rhadinovirus* | BoHV-4 | AF318573 |
| *Bovine herpesvirus 4* | *Rhadinovirus* | BoHV-4 | JN133502 |
| *Bovine herpesvirus 4* | *Rhadinovirus* | BoHV-4 | KC999113 |
| *Sus barbatus rhadinovirus 1* | *Rhadinovirus* | SbarRHV-1 | AY177147 |
| *Babyrousa babyrussa rhadinovirus 1* | *Rhadinovirus* | BbabRHV-1 | AY177146 |
| *Saimiriine herpesvirus 2* | *Rhadinovirus* | SaHV-2 | X64346 |
| *Pan troglodytes rhadinovirus 1* | *Rhadinovirus* | PtroRHV-1 | AY138585 |
| *Bandicota indica rhadinovirus 4* | *Rhadinovirus* | BindRHV -4 | EF128043 |
| *Ateline herpesvirus 3* | *Rhadinovirus* | AtHV-3 | AF083424 |
| *Human herpesvirus 8* | *Rhadinovirus* | HHV-8 | KSU75698 |
| *Macacine herpesvirus 5* | *Rhadinovirus* | McHV-5 | AF210726 |
| *Apodemus flavicollis rhadinovirus 1* | *Rhadinovirus* | AflaRHV-1 | DQ821580 |
| *Murid herpesvirus 4* | *Rhadinovirus* | MuHV-4 | AF105037 |
| *Human herpesvirus 4* | *Lymphocryptovirus* | HHV-4 | AJ507799 |
| *Macacine herpesvirus 4* | *Lymphocryptovirus* | McHV-4 | NC_006146 |
| *Ateles paniscus lymphocryptovirus 1* | *Lymphocryptovirus* | ApanLCV-1 | AY139028 |
| *Tapirus terrestris gammaherpesvirus 1* | *Gammaherpesvirus* | TterGHV-1 | AF141887 |
| *Panthera leo gammaherpesvirus 1* | *Gammaherpesvirus* | PleoGHV-1 | DQ789370 |
| *Alcelaphine herpesvirus 1* | *Macavirus* | AlHV-1 | NC_002531 |
| *Caprine herpesvirus 2* | *Macavirus* | CpHV-2 | AF283477 |
| *Ovine herpesvirus 2* | *Macavirus* | OvHV-2 | AY839756 |
| *Equid herpesvirus 2* | *Percavirus* | EHV-2 | HQ247790 |
| ***Subfamily: Alphaherpesvirinae*** |  |  |  |
| *Bovine herpesvirus 1* | *Varicellovirus* | BoHV-1 | AJ004801 |
| *Bovine herpesvirus 5* | *Varicellovirus* | BoHV-5 | AY261359 |
| *Suid herpesvirus 1* | *Varicellovirus* | SuHV-1 | NC_006151 |
| *Bovine herpesvirus 2* | *Simplexvirus* | BoHV-2 | AF181249 |
| *Human herpesvirus 1* | *Simplexvirus* | HHV-1 | NC_001806 |
| *Human herpesvirus 2* | *Simplexvirus* | HHV-2 | NC_001798 |
| *Macacine herpesvirus 1* | *Simplexvirus* | McHV-1 | NC_004812 |
| *Gallid herpesvirus 1* | *Iltovirus* | GaHV-1 | NC_006623 |
| *Psittacid herpesvirus 1* | *Iltovirus* | PsHV-1 | NC_005264 |
| *Gallid herpesvirus 2* | *Mardivirus* | GaHV-2 | NC_002229 |
| *Gallid herpesvirus 3* | *Mardivirus* | GaHV-3 | NC_002577 |
| *Melegrid herpesvirus 1* | *Mardivirus* | MeHV-1 | NC_002641 |
| ***Subfamily: Betaherpesvirinae*** |  |  |  |
| *Human herpesvirus 5* | *Cytomegalovirus* | HHV-5 | NC_006273 |
| *Suid herpesvirus 2* | *Cytomegalovirus* | SuHV-2 | AF268040 |
| *Human herpesvirus 6* | *Roseolovirus* | HHV-6 | NC_001664 |
| *Human herpesvirus 7* | *Roseolovirus* | HHV-7 | NC_001716 |
| *Murid herpesvirus 2* | *Muromegalovirus* | MuHV-2 | NC_002512 |
