# Supplementary material for: Whole Genome Sequencing of a Canadian Bovine Gammaherpesvirus 4 Strain and the Possible Link between the Viral Infection and Respiratory and Reproductive Clinical Manifestations in Dairy Cattle
Source: Front Vet Sci. 2017 Jun 16;4:92. doi: 10.3389/fvets.2017.00092 (PMC5472674; doi:10.3389/fvets.2017.00092)
Supplement: Supplementary file 2 [file Table_2.DOCX]

**Supplemental Table 2.** List of the 27 sequences used in the TK gene molecular phylogeny of bovine herpesvirus.

| **Isolates** | **Accession** |
| --- | --- |
| BoHV-4-FMV (FMV) | KC999113 |
| 66-p-347 | AF318573 |
| v.test | JN133502 |
| MGA1075 | EU244700 |
| MGA514 | EU244699 |
| MGA696 | EU244698 |
| MGArom | EU244697 |
| 86_06 | AB035517 |
| Movar_33_63 | AB035516 |
| B11_41 | AB035515 |
| DN599 | JQ838062 |
| 10_154 | JQ838061 |
| 09_759 | JQ838060 |
| 09_508 | JQ838059 |
| 08_467 | JQ838058 |
| 09_465 | JQ838057 |
| 09_227 | JQ838056 |
| 08_476 | JQ838055 |
| 08_433 | JQ838054 |
| 08_415 | JQ838053 |
| 08_404 | JQ838052 |
| 08_362 | JQ838051 |
| 08_263 | JQ838050 |
| 08_209 | JQ838049 |
| 07_759 | JQ838048 |
| 07_568 | JQ838047 |
| 07_435 | JQ838046 |
